# Diphtheria

**Published:** 1862-01

**Authors:** 


### DIPHTHERIA.

In the *Ohio Medical and Surgical Journal* for August, Dr. E. L. Plympton, of Centreville, Ohio, has an article upon diphtheria, from which we make an extract or two, and upon which we propose to base a remark Dr. Plympton's experience early led him to the following conclusions:

"1st. That diphtheria is as much a blood disease as small pox. 2d. That it should be treated with such hematic remedies as have a tendency to correct this morbid condition. 3d. That the treatment, to be effective, must be commenced either before or early in the active stage of the disease, and that it is useless to waste much time or trouble in treating the local affection of the throat. Acting upon these conclusions, I have, in every case, placed my principal reliance upon some combination of chlorine, and mainly upon the chlorate of potash, commencing usually with a mild mercurial cathartic, oftener using the hyd. c. creta than any other. To a child five years of age, I give enough of the saturated solution of the chlorate to contain three or four grains, and repeat the dose every three hours during the career of the disease; and to patients older or younger, in relative proportions. When the child is capable of gargling, I have him use one mouthful as a gargle, for the purpose of washing out the loose excretions of the throat, and then immediately swallow the prescribed dose. I have repeatedly penciled the throat with nitrate of silver and tincture of iodine without any very satisfactory results."

In regard to removing the exudative membranes our opinions have been previously expressed; and, though we differ in this regard with most authorities, we find support in our friend's opinion. He says:

"I can see no more philosophy in removing the membranous exudation with the expectation of mitigating or cutting short the disease, than I can in removing the pestules of variola with the expectation of safely terminating that disease. The one is as much an element of systemic disease as the other. If the diphtherial exudation be in the air-passages below the epiglottis, the caustic swab will stand a poor chance of removing it in season to save the patient; if the exudation be above, it will not be much in the way of his recovery."

This opinion is almost exactly what we have expressed on a former occasion.

At the head of all remedial agents in diphtheria, Dr. Plympton places the chlorate of potash, and he says:

"I not only have faith in it as a *curative* remedy, but as a *prophylactic.*"

Bearing upon the subject of its prophylactic powers, we will quote an idea from Dr. S. H. Smith, of New York. In the *American Medical Times*, for June 8th, speaking of the chlorate of potash in *typhoid fever*, he says:

"I have settled down into the conviction that its greatest value in such cases is as a prophylactic. Given in conjunction with quinine and mild aperients, I have seen it repeatedly stave off attacks of fever during epidemics, even of 'Irish emigrant' or 'ship' fever; the premature laying aside of the medicine being followed by immediate return of the threatening symptoms, again to be dispersed by a recurrence to its use."

We are inclined to attribute the prophylactic powers of this combination largely to the quinine, yet we give the opinion of Dr. Smith as it stands. It must be admitted that there are some points of strong resemblance between typhoid fever and diphtheria.

We have used the chlorate of potash in every case of diphtheria that has come under our observation, and we must say we are not satisfied as to its curative properties. Certain are we, that we have never lost a case in which the chlorate of potash was not liberally used from the beginning. Though it may be a valuable remedy, *it is not capable of saving all cases*, even when aided and supported by other appropriate remedies.

Our readers may remember that in the *Reporter* for September 14th, we referred to the opinions of Dr. N. E. Jones, of Circleville, Ohio, as published in the *St. Louis Medical and Surgical Journal* for May. He regards belladonna as a valuable remedy in diphtheria, and gives the following as his conclusions:

"1st—Belladonna given to intoxication arrests membranous exudation.

2d—Given early, in the febrile stage, cures by resolution.

3d—Causes softening and detachment of exudation in an unusually short space of time."

Upon this point, in the *St. Louis Medical and Surgical Journal* for July, Dr. E. W. White remarked:

"Exudations are respectively euplastic, caeoplastic, or aplastic. In the case of croup, the formation may be plastic, highly fibrinous; in scarlatina, almost entirely aplastic, sero-albuminous, and tending to putrefaction.—Diphtheria appears to rank between the two, the effusion being either fibrinous, when the attack is acute and sthenic, or fibro-albuminoid; or, indeed, if slow, insidious, and asthenic in its approach, the effusion may be sero-albuminoid. Now, we cannot understand how belladonna can exert any beneficial effect in such diphtheria, where all anti-phlogistics or sedatives are injurious. *We need stimulants from the beginning.* If it be true that malignant diphtheria, scarlatina, and croup are blood diseases, a narcotic is certainly not indicated—alteratives are needed. *I think iodide of potassium has probably the highest claim.* It has always been used with benefit in the sloughing, mercurial sore throat, a condition almost identical with that of diphtheritic ulceration. Mercury would be, for this reason, a bad remedy, especially after the acute stage had passed. Iron and alkalies are the great constitutional means. Diphtheria is a true blood disease, and I would use stimulants to elevate the nervous power, while I would use alteratives and tonics to cure the blood disease. It would appear, therefore, irrational empyricism to administer belladonna '*to intoxication*' in these diseases."

Of belladonna in diphtheria we cannot speak favorably; and, th ugh the chlorate of potash may be a valuable remedy, there are many cases to which it is not adequate to the cure.

Our brother-in-law, Dr, L. V. Axtell, of Jamestown, N. Y., whose experience is quite large in this disease, speaks very highly of *arsenic* in diphtheria. In adults, he would give *Fowler's solution* in ten-drop doses, and repeat every *four* or *six* hours during the disease. He says the proportion of deaths to his whole number of cases has been greatly diminished since he commenced the administration of this remedy. He also believes that the fetor of the breath has been greatly diminished under this kind of medication. It is proper to observe that he has not neglected ordinary instrumentalities—quinine, iron, chlorate of potash, etc. have been used as heretofore.

Theoretically, we should expect benefit from it. A remedy so powerfully antiseptic, and tonic besides, should be of service in a disease so strongly septic as is diphtheria in its severer forms, as well as in some other difficulties of a similar character.

We have used the remedy in our last ten cases, and we were rapidly acquiring a favorable opinion of it, when we suddenly lost two cases, both in one day. They were, however, terrible cases, septic to the last degree, and it was probably no fault of the medicine that the patients died. In so severe a disease as diphtheria, it is not probable that a specific will be found. Dr. Axtell and his partner have great confidence in arsenic, and we certainly hope much from it. In this locality, a type of the disease has prevailed more putrid in its tendencies and rapidly gangrenous than any we have seen described in all our reading. It has been our good fortune to lose but one in ten. In view of all the circumstances, we think this speaks well for the remedies employed. When we hear of physicians having hundreds of cases, and losing none—using only simple remedies—we know they have not seen the disease at all, or at least not in its severer forms.

Dr. White, of St. Louis, suggests the *iodide of potassium* as probably the most important alterative in diphtheria. We remember having seen the same suggestion before, but do not now call to mind by whom it was made. We have never given this article a trial in this disease, and can say nothing experimentally.—*Medical and Surgical Reporter.*

